# Evaluation of Surgical Protocols for Speech Improvement in Children with Cleft Palate: A Systematic Review and Case Series

**DOI:** 10.3390/bioengineering12080877

**Published:** 2025-08-14

**Authors:** Angelo Michele Inchingolo, Gianna Dipalma, Paola Bassi, Rosalba Lagioia, Mirka Cavino, Valeria Colonna, Elisabetta de Ruvo, Francesco Inchingolo, Giuseppe Giudice, Andrea Palermo, Alessio Danilo Inchingolo

**Affiliations:** 1 Department of Interdisciplinary Medicine, University of Bari “Aldo Moro”, 70124 Bari, Italy; angeloinchingolo@gmail.com (A.M.I.); gianna.dipalma@uniba.it (G.D.); paola.bassi@uniba.it (P.B.); rosalba.lagioia@uniba.it (R.L.); mirka.cavino@uniba.it (M.C.); valeria.colonna@uniba.it (V.C.); studio.deruvo@libero.it (E.d.R.); alessiodanilo.inchingolo@uniba.it (A.D.I.); 2 Unit of Plastic and Reconstructive Surgery, Department of Precision and Regenerative Medicine and Jonic Area, University of Bari, 11, Piazza Giulio Cesare, 70124 Bari, Italy; giuseppe.giudice@uniba.it; 3 Department of Experimental Medicine, University of Salento, 73100 Lecce, Italy; andrea.palermo@unisalento.it

**Keywords:** cleft palate, speech outcomes, palatoplasty, cleft palate surgery timing

## Abstract

Background: This systematic review investigates how different surgical techniques influence speech outcomes in children with cleft palate, focusing on the effectiveness of key palatoplasty methods and the timing of surgery on vocal function. Methods: A thorough search of the PubMed, Scopus, and Web of Science databases was conducted for studies published between 2014 and 2024, including clinical research reporting speech results after palatal repair, with bias assessed using the ROBINS tool. Additionally, two clinical cases are presented to demonstrate the practical application of the surgical approaches. Results: Analysis of fourteen studies revealed that modified Z-plasty and V-Y procedures enhance soft palate mobility and reduce hypernasality, although they require advanced surgical skills. Early closure of the hard palate, performed within the first year of life, was linked to improved consonant articulation compared to later surgeries. No significant differences were found between single-stage and two-stage repairs, but surgeon experience emerged as a crucial factor influencing outcomes. Conclusions: Overall, both the surgical technique selected and the timing of intervention play important roles in optimizing speech development in children affected by cleft palate.

## 1. Introduction

**Figure 1:**
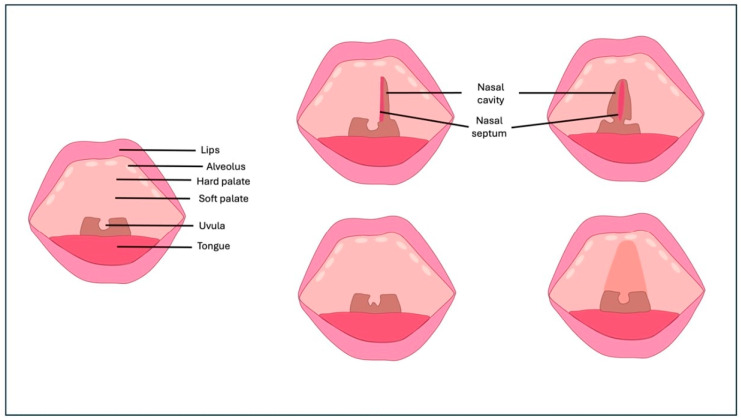
Different subtypes and clinical forms of cleft palate.

Orofacial clefts (OFCs) are one of the most important congenital malformations [1,2,3,4,5]. The overall prevalence of these conditions varies from approximately 1:700 to 1.5:1000 births, with marked differences according to geographical area, ethnicity, and sex: cleft lip (CL) is more common in males, while isolated cleft palate (CP) is more common in females, and transmission is autosomal dominant [6,7,8,9,10]. In addition, about 30% of cases are associated with genetic syndromes, including Van der Woude syndrome, 22q11.2 deletion, and Pierre Robin sequence, while the remainder are nonsyndromic manifestations, the result of multifactorial interactions between genetic and environmental elements [11,12,13,14,15,16,17,18]. Cleft lip and palate (CL/P) can be classified into different forms depending on its extent and severity (Figure 1).

CL can be microform, incomplete, or complete. The microform form presents as a simple incision or depression in the vermillion, while the incomplete form also involves the orbicularis muscle but does not extend to the base of the nose [19,20,21,22]. The complete form extends to the nostril, resulting in an altered insertion of the orbicularis muscle.

CP may be limited to the soft palate or may involve the hard palate. In the first case, the cleft involves the entire palate from the incisor canal to the uvula, so that the base of the nasal septum can be seen offset from the palatine laminae on the midline [23,24,25,26,27,28,29,30,31,32,33,34,35]. In cleft palate, there is no structural change in the upper jaw, but there is a passage for food and air into the nose [36,37,38,39,40,41,42,43,44,45,46,47,48,49,50,51,52]. In the most extensive forms, both the primary and secondary palates are affected, resulting in direct communication between the oral and nasal cavities [53,54,55,56,57,58,59,60,61,62,63].

The palate plays a crucial role in phonation, providing the separation between the oral and nasal cavities and allowing the intraoral pressures necessary to produce sounds, particularly high-pressure sounds, to be generated [48,51,64,65,66,67,68,69,70,71,72,73,74,75].

The palatal muscles, particularly the palatal elevators, are essential for the elevation and correct positioning of the soft palate during swallowing and phonation [76,77,78,79,80].

Adequate function of this complex mechanism is essential to avoid excessive air release through the nasal cavity, a condition that manifests clinically as velopharyngeal insufficiency (VPI) with hypernasality, nasal emission, and articulatory disorders.

Prenatal diagnosis of cleft lip and palate is possible by morphological ultrasound, which is performed between the 18th and 20th weeks of pregnancy. Two-dimensional (2D) ultrasound can detect the presence of CL, while three-dimensional (3D) ultrasound offers greater diagnostic accuracy, allowing a more detailed assessment of the orofacial structures. Early identification of the malformation allows parents and the medical team to be adequately prepared for a tailored postnatal treatment plan [81,82,83].

Surgical treatment of CP, usually performed within the first year of life, aims to restore the correct anatomy and promote the development of a functional velopharyngeal mechanism. Several techniques have been developed over the years, such as linear closure with intravelar veloplasty (IVVP), Sommerlad’s technique, Furlow’s double-opposing Z-plasty technique, and two-stage procedures involving early repair of the soft palate followed by correction of the hard palate. The choice of surgical method depends on the extent of the deformity, the timing of the operation, and the experience of the multidisciplinary team, always bearing in mind the delicate balance between functional restoration and the potential impact on jaw growth.

Alongside surgery, the importance of speech therapy rehabilitation for subsequent speech development is clear [84]. Coordination of the palatal velopharyngeal muscles and modulation of intraoral pressure are critical for articulation and resonance quality. The use of standardized assessment tools, such as the percentage of correct consonants (PCC) and specific scales of velopharyngeal function, makes it possible to monitor speech development and highlight the need for targeted rehabilitation interventions to correct any functional deficits [85,86].

In conclusion, the management of CP is a complex challenge that requires a thorough understanding of the embryological and anatomical dynamics, a tailored surgical strategy, and ongoing logopedic support. The integration of these skills in a multidisciplinary approach is essential to restore velopharyngeal function and promote the development of clear and articulate speech, thereby improving the quality of life of patients from the earliest years of development [87,88].

In light of these considerations, the present study aims to evaluate the efficacy of surgical treatment of CP from a linguistic point of view, by analyzing whether and to what extent improvements in phonetic production and velopharyngeal function have occurred in operated patients [89,90].

## 2. Materials and Methods

### 2.1. Protocol and Registration

This systematic review adhered to the PRISMA (Preferred Reporting Items for Systematic Reviews and Meta-Analyses) guidelines. The protocol was registered on PROSPERO (The International Prospective Register of Systematic Reviews) under the following reference number: 1005298. In addition to the review, this article also presents a series of clinical cases focusing on surgical treatments for cleft palate and, to a lesser extent, cleft lip. Patients who underwent surgical intervention were examined at the University of Bari. The analysis highlights the impact of different surgical techniques and the timing of intervention on treatment outcomes. Furthermore, two case reports have been included to provide additional clinical insight:

- Case 1: Two 6-month-old patients with cleft lip underwent cleft repair surgery using a modified Millard technique. At one-year follow-up, good-quality scars without retraction were observed, indicating a successful outcome.
- Case 2: A 6-month-old patient with unilateral cleft of the soft palate underwent cleft repair surgery using intravelar veloplasty. Postoperative evaluation confirmed complete closure of the soft palate and adequate velopharyngeal competence.

These case reports complement the systematic review by illustrating the practical application of different surgical techniques and their impact on speech outcomes in children with cleft conditions.

### 2.2. Search Strategy

A systematic literature search was conducted in Scopus, Web of Science (WoS), and PubMed using the following keywords: “Cleft palate” AND “Speech outcomes”.

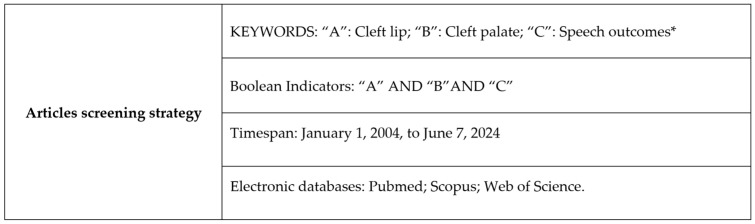



### 2.3. Inclusion and Exclusion Criteria

The search was limited to English-language articles published within the last ten years (2014–2024) (Table 1). Studies were selected through a double-blind review process based on the following inclusion criteria: (1) research involving human subjects and (2) clinical studies, randomized controlled trials, observational studies, and speech outcomes as a primary endpoint. Exclusion criteria included review articles, meta-analyses, animal studies, and in vitro research, as well as articles lacking free full-text access. The following process was used to construct the PICO model:

Population (P): Children with cleft palate undergoing surgical intervention for speech improvement.
Intervention (I): Different surgical protocols for cleft palate correction, including the following:
  - Sommerlad technique;
  - Von Langenbeck palatoplasty;
  - Furlow’s double-opposing Z-plasty;
  - Modified V-Y palatoplasty;
  - Two-stage palatoplasty (IVVP and Von Langenbeck);
  - Vomeroplasty.
Comparison (C): Comparison between different surgical techniques or timing of interventions:
  - Single-stage vs. two-stage palatoplasty;
  - Early vs. delayed hard palate closure;
  - Techniques with mucosal graft vs. without mucosal graft.
Outcome (O): Speech outcomes including the following:
  - Velopharyngeal insufficiency (VPI);
  - Nasal resonance;
  - Consonant competence (PCC);
  - Speech intelligibility;
  - Postoperative complications (oronasal fistulas, wound dehiscence);
  - Need for secondary surgical interventions.

### 2.4. Data Screening and Selection

The screening process involved an initial review of titles and abstracts, followed by a full-text assessment of potentially eligible studies. Articles deemed irrelevant were excluded. Any disagreements between reviewers were resolved through discussion, and in cases of persistent discrepancies, a third reviewer (F.I.) was consulted to reach a consensus. 

## 3. Results

**Figure 2:**
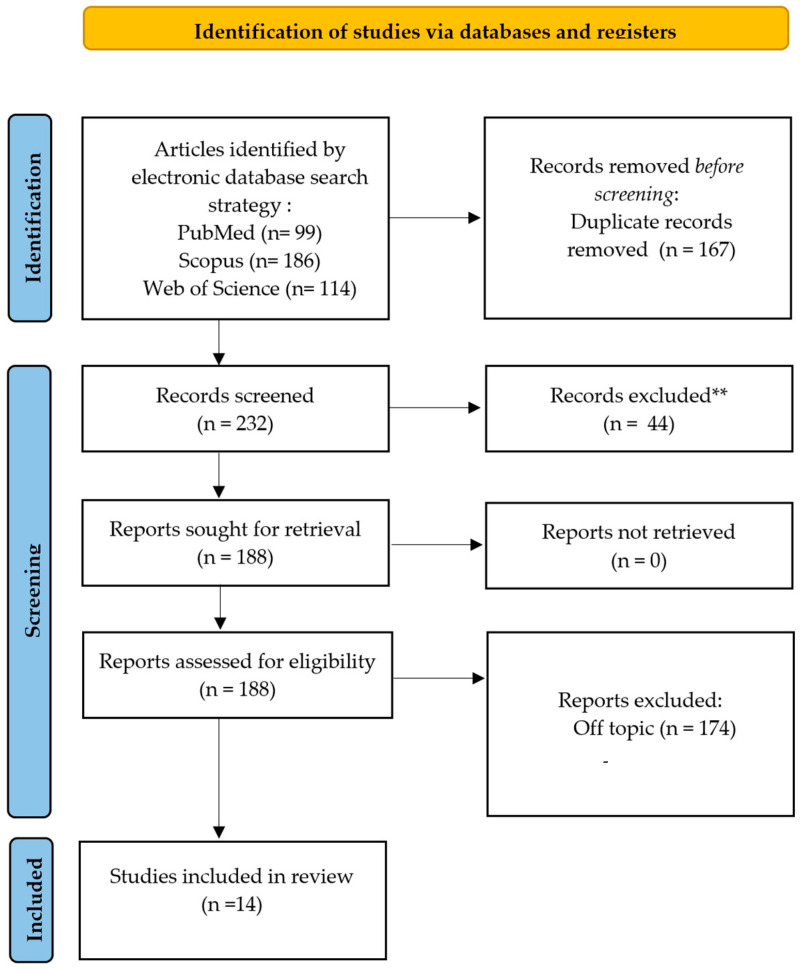
PRISMA flow chart.

The database search retrieved a total of 399 articles, distributed as follows: PubMed (99), Web of Science (114), and Scopus (186). After eliminating 167 duplicate entries, 232 articles remained for screening. Based on title and abstract evaluation, 174 articles were excluded as off-topic, 23 due to restricted access, and 21 as review articles. No studies were found to involve animal models or in vitro research. Following full-text screening, 14 studies met the inclusion criteria and were selected for qualitative analysis. The selection process is summarized in Figure 2. Each study’s findings are presented in Table 1.

### Quality Assessment

**Figure 3:**
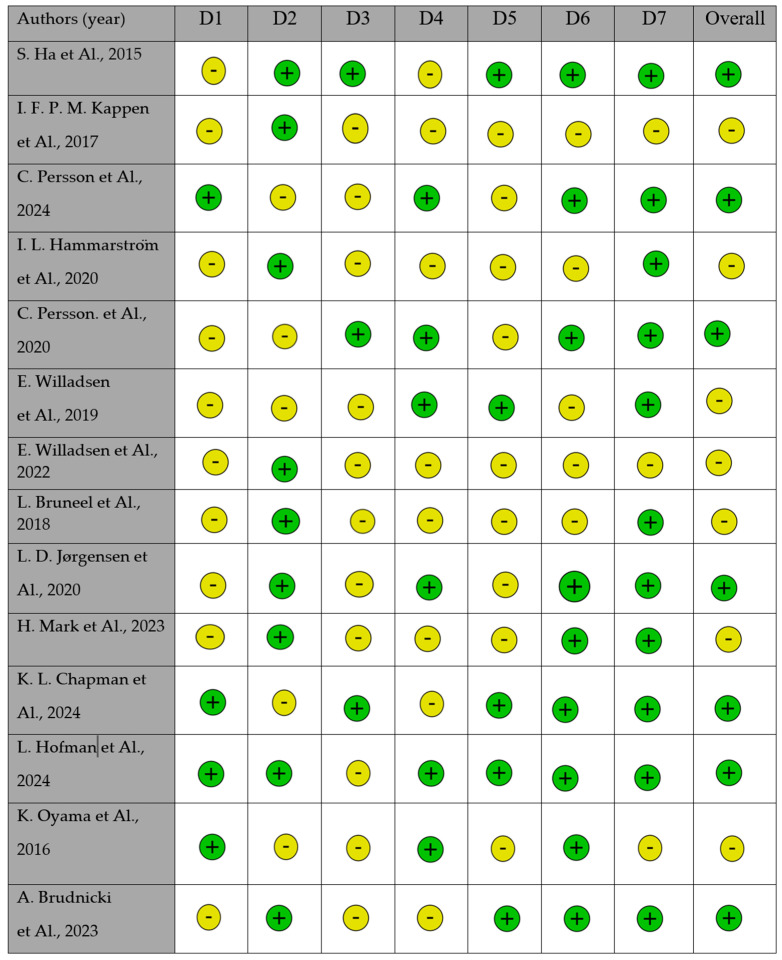

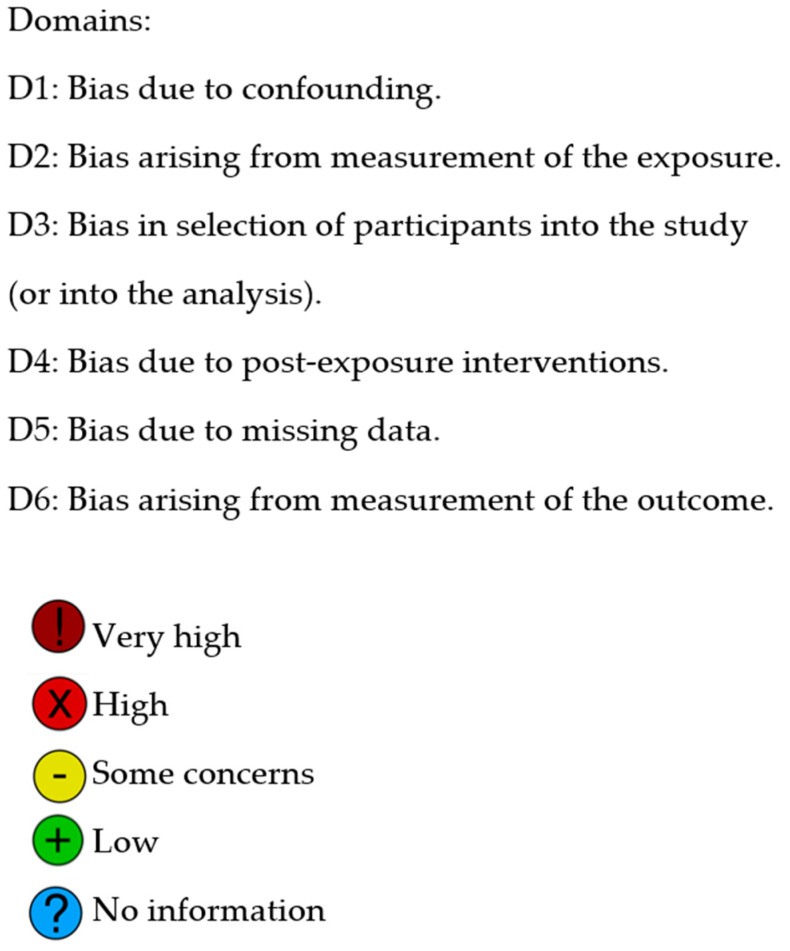
Risk of bias by Robin tool [76,77,91,92,93,94,95,96,97,98,99,100,101,102].

The ROBINS tool was applied to evaluate the risk of bias in non-randomized studies comparing the effects of different interventions on health outcomes (Figure 3). Two independent reviewers (R.L. and P.B.) assessed the bias levels for each of the seven ROBINS domains. The risk-of-bias analysis revealed that most studies presented some concerns or low risk in domains related to exposure measurement (D2) and outcome measurement (D6). However, certain studies exhibited a high risk of bias regarding missing data (D5) and participant selection (D3), particularly when the inclusion criteria were not clearly described or when small sample sizes were analyzed.

More recent studies (2023–2024) tend to show a lower risk of bias, indicating an improvement in methodological quality compared to earlier publications. 

Any disagreements were resolved through discussion, with the involvement of a third reviewer (F.I.) when necessary.

**Case Series**

**Figure 4:**
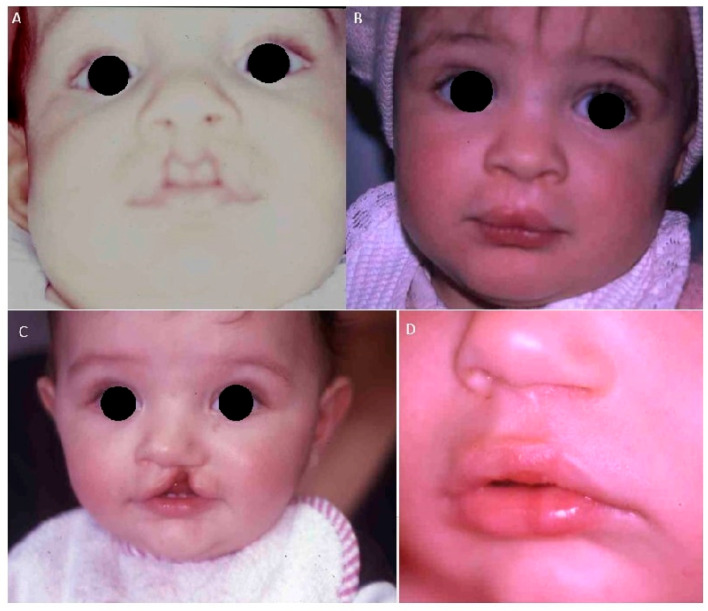
(**A**–**C**) Six-month-old patient affected by bilateral (**A**) and monolateral (**C**) cleft lip treated with modified Millard technique at six months. (**B**–**D**) Postoperative view at 1-year follow-up. Scars appear of good quality with no retraction.

**Cases 1–2.** Two 6-month-old patients with cleft lip underwent cleft repair surgery using a modified Millard technique. Good-quality scars without retraction were found at follow-up (Figure 4).

**Figure 5:**
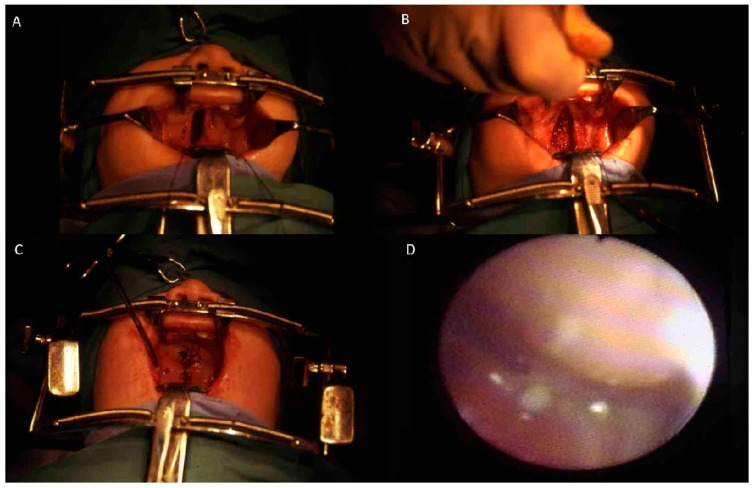
(**A**) A 6-month-old patient affected by soft palate unilateral cleft. An intravelar veloplasty was planned. (**B**) Intraoperative view showing the closure of the palate muscolar layer. (**C**) Intraoperative view at the end of the procedure showing the complete closure of the soft palate. (**D**) Nasopharyngoscopy shows adequate velopharyngeal competence.

**Case 3**. A 6-month-old patient with unilateral cleft of the soft palate underwent cleft repair surgery by means of intravelar veloplasty. At the end of surgery, complete closure of the soft palate and adequate velopharyngeal competence were found (Figure 5).

## 4. Discussion

### 4.1. CP and Speech

The aim of this systematic review is to analyze the literature of the last 10 years on surgical protocols for the treatment of children with CP, with a focus on identifying the factors that lead to an improvement in patients’ speech. OFCs cause severe morphological changes in affected patients, as well as severe functional deficits in sucking and swallowing in neonates, and subsequently in phonation, chewing, and breathing. There is a clear correlation between CP and speech disorders, so it is important to assess the speech of the patient with cleft palate within the first six months of life and to monitor it throughout adolescence.

Phonation and swallowing functions depend on the muscles of the soft palate (palatine elevator, palatine tensor, palatopharyngeal, palatoglossus, and uvula muscles), which, in a person without CP, provide velopharyngeal closure. In particular, during phonation and swallowing, these muscles move the soft palate superiorly and posteriorly to bring it closer to the pharyngeal wall and achieve closure between the nasal and oral cavities. In a patient with CP, speech disorders are due to the abnormal orientation and muscle insertion, particularly of the palatal elevator muscle on each side, which instead of inserting transversely at the level of the median palatine aponeurosis, inserts longitudinally at the level of the posterior border of the palatine bone [103,104,105]. In addition, these patients have impaired sphincter function of the palatoglossus, palatopharyngeus, and superior constrictor muscles. VPI, i.e., the inability of the soft palate and posterior and lateral pharyngeal walls to close the communication between the oral and nasal cavities during phonation, causes children with CP to have a nasal-like speech due to nasal air escaping during speech. Nasopharyngoscopy is indicated to assess velopharyngeal function and determine the need for surgical correction [106,107].

### 4.2. Palatoplasty Techniques and Speech Outcomes

The treatment of OFCs requires a multidisciplinary approach involving the surgeon, otolaryngologist, orthodontist, speech therapist, and phoniatrist. Of fundamental importance is the correction of any functional deficits of the soft palate to ensure correct phonation.

**Figure 6:**
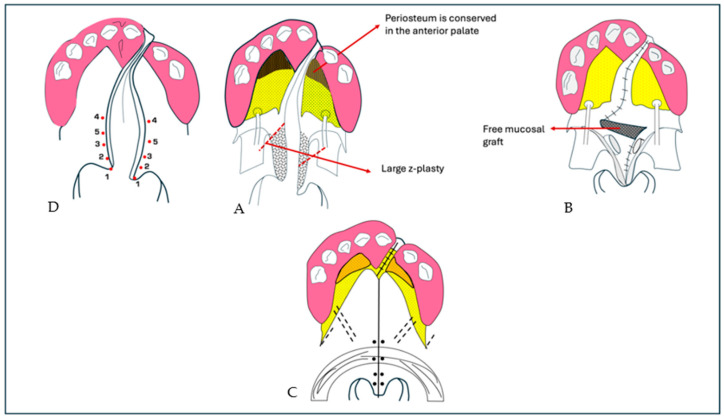
Surgical procedures for MG group palatal repair of UCLP. The incision line and anatomical landmarks are depicted in the figure. (**A**) An extended Z-plasty on the nasal side and elevation of the palatal flaps preserving the periosteum in the anterior and lateral portions of the hard palate (dotted line). (**B**) A nasal mucosal graft that is free. (**C**) A levator sling is produced by symmetrical muscle reconstruction while referring to (**D**) the landmarks of the anatomical lines.

The main aim of palatoplasty, i.e., surgical correction of CP, is to normalize the patient’s phonatory function without compromising maxillofacial growth. The operation involves the correct repositioning of the palatal muscles to ensure the exact function of the soft palate (Figure 6).

Several authors have conducted studies aimed at identifying the surgical technique for the correction of CP that could ensure better lingual outcomes.

Sommerlad’s palatoplasty is one of the surgical techniques used to treat CP; it aims to restore palatal morphology and velopharyngeal function. Sommerlad’s technique involves repositioning the muscles of the soft palate, particularly the elevator muscle of the palatine veil, and closing the palatal defect with mucoperiosteal flaps.

In a 2024 retrospective study, Hofman et al. evaluated the long-term speech outcomes of CP patients who underwent correction using Sommerlad’s technique [100]. Their findings revealed that 52.7% of patients developed VPI, and 49.8% required speech correction. Although the age of patients (average 10 months) did not significantly affect the results, larger cleft sizes (>10 mm) and postoperative complications, such as wound dehiscence and oronasal fistulas (FON), were associated with higher rates of VPI and the need for further speech intervention. Thus, while Sommerlad’s technique is effective for CP repair, the speech outcomes heavily depend on defect size and complications.

Bruneel et al. (2018) compared the speech outcomes of patients treated with Sommerlad palatoplasty against a control group of children without CP [96]. The study, conducted at Ghent University Hospital, found that, compared to the controls, Sommerlad-treated patients exhibited persistent issues with intelligibility and mild to moderate hypernasality. Nasal resonance was measured using the Kay Pentax II nasometer, allowing for the calculation of the Nasality Severity Index (NSI).

Palatoplasty using the von Langenbeck technique involves making incisions along the edges of the CP and on the oral mucosa, then peeling off flaps to suture the palatal muscles, and then repositioning the flaps.

A 2017 study by Kappen et al. examined speech outcomes in patients with unilateral complete cleft lip and palate (UCLP) treated with a two-stage palatoplasty [92]. Treatment included surgical closure of the soft palate at 7.8 months by IVVP and of the hard palate at 40 months by the Von Langenbeck technique, and pharyngoplasty in the case of VPI. IVVP allows repair of the soft palate by suturing the muscles of the palatal veil. The majority of treated patients showed good long-term speech intelligibility, but articulation disorders and mild hypernasality were also found to reduce speech quality. Factors such as scarring and fistulas negatively affected speech outcomes, suggesting the need for improved surgical protocols.

Furlow’s double-opposing Z-plasty technique involves the placement of two triangular flaps with opposing bases, forming a Z at 60° angles. These flaps are dissected, rotated, and sutured.

Chapman et al., through the CORNET project funded by the National Institutes of Health, are investigating the speech outcomes at 36 months following two different palatoplasty procedures: Furlow’s Z-plasty and IVVP [99]. Although data collection has been delayed due to the COVID-19 pandemic, this study continues to evaluate surgical techniques and speech interventions for children with CP.

Double-flap palatoplasty is a surgical technique in which extended incisions up to the uvula are made along the margins of the cleft; bilateral release incisions are made to reduce tension. Once the flaps have been divided to full thickness, the palatal elevator muscles are repositioned horizontally and sutured.

A retrospective study by Ha et al. assessed postoperative outcomes for CP patients who underwent palatoplasty with Furlow’s double-opposite Z-plasty, double-flap palatoplasty, IVVP, or von Langenbeck techniques [91]. The results showed that most patients experienced FONs, VPI, and speech disorders.

In modified V-Y palatoplasty, an initial ‘V’-shaped incision is made at the junction of the hard and soft palate. Once the flaps have been mobilized, another incision is made to form a ‘Y’. At this point the flaps are pulled and sutured to close the defect. Oyama et al. compared postoperative speech outcomes in CP patients treated with modified V-Y palatoplasty, with or without mucosal grafting [101]. Their results indicated that palatoplasty with mucosal grafting produced significantly better speech outcomes in terms of hypernasality, nasal emission, articulation, and nasal resonance compared to the non-grafted technique, which was similar to the speech of children without CP. Cephalometric analysis also revealed a longer and thinner soft palate with improved phonatory movement in mucosa-transplanted patients.

Palatoplasty using a double-layer vomeroplasty technique often uses the vomer as a support for the repair. This technique involves the closure of two layers, a deeper muscular layer to ensure the restoration of function and a more superficial layer of mucosa to promote healing.

Brudnicki et al. evaluated speech outcomes and the need for secondary surgeries in UCLP patients undergoing early (before 6 years) or late (after 6 years) alveolar bone grafting [102]. Their results indicated that primary palatal closure using a double-layer vomeroplasty technique and early alveolar bone grafting provided better speech intelligibility and reduced the need for secondary surgeries compared to late alveolar bone grafting.

In summary, the studies reviewed emphasize the critical role of surgical technique choice in determining speech outcomes in CP patients. Techniques such as Sommerlad’s and von Langenbeck’s palatoplasty are effective in correcting CP but may lead to persistent speech problems when complications like FON or VPI arise. More complex techniques, such as Furlow’s Z-plasty and modified V-Y palatoplasty with mucosal grafting, though requiring greater surgical skill, provide better long-term speech outcomes, including improved soft palate mobility, reduced hypernasality, and enhanced intelligibility.

### 4.3. Timing of CP Treatment and Speech Outcomes

Surgical repair of the hard and soft palate ensures better phonatory function if performed before the child’s speech development stage, which usually occurs at 9–12 months of age. Most surgeons repair the palate in one operation at 12 months, while others repair the soft palate at 3–6 months, at the time of primary lip repair, and the hard palate before 12–15 months. Although the timing of treatment differs between the different schools of thought, the common goal is to provide treatment aimed at functional and morphological restoration of the face, while respecting the patient’s growth potential.

Over the years, several authors have carried out studies aimed at understanding which surgical protocol could allow the child to recover a better phonatory capacity, focusing on the timing of treatment in children with CP.

In 2023, Marka et al. compared two protocols for treating UCLP patients: early soft palate closure at 6 months and hard palate closure at 3 years versus late hard palate closure at 8 years [98]. The study found that patients who underwent hard palate closure at 3 years had improved oral articulation and intelligibility, whereas late repair enhanced palatal growth.

The Scandcleft project is a multicenter study of the results of three parallel randomized controlled trials (RCTs) involving ten teams of surgeons from five countries (Denmark, Finland, Norway, Sweden, UK). The aim was to compare different surgical treatment protocols for 448 children with UCLP and to analyze their speech and language outcomes, focusing on 5-year and 10-year outcomes. In particular, the study investigated the effect of the timing of hard palate closure on velopharyngeal function and consonantal competence as measured by PCC, i.e., the number of correctly produced consonants divided by the total number of consonants predicted in spontaneous speech. The results of children with UCLP were compared with those of children without UCLP of the same age.

In 2019, Willadsen et al. reported the results from Trial 1 of the Scandcleft project, comparing two protocols for 143 children with UCLP [76]. Group A underwent soft palate closure at 3–4 months and hard palate closure at 12 months, while group B had soft palate repair at 3–4 months and hard palate repair at 36 months. No significant differences were observed in VPI prevalence, but group A had a significantly better PCC score. Jørgensen and Willadsen further analyzed data from Trial 1, focusing on Danish children, revealing that those undergoing early surgery demonstrated significant improvements in PCC scores, except for the /k/ sound.

In 2020, Hammarström et al. published the results from Trial 2 of the Scandcleft project, assessing velopharyngeal function and PCC scores in patients who underwent one-stage or two-stage palatal closure [94]. The results showed that only 60–70% of the children had normal speech at the age of 5 years. Velopharyngeal function was similar between the groups, but the PCC score was significantly higher in children without CP; only 27% of patients undergoing palatal closure achieved an age-appropriate PCC. The authors concluded that the choice between one-stage or two-stage palatal surgery does not significantly influence language outcomes; however, the language spoken and the surgeon’s experience may play a role.

The results from Trial 3 of the Scandcleft project were published by Persson et al. in 2020 [95]. The study analyzed language outcomes in 5-year-old children with UCLP undergoing two different two-stage surgical protocols: group 1 underwent lip and soft palate closure at 3–4 months and hard palate closure at 12 months, while group 2 underwent lip and hard palate closure at 3–4 months and soft palate closure at 12 months. No statistically significant differences were found between the two groups with regard to velopharyngeal function, while group 2 showed significantly better PCC scores than group 1, which revealed more errors in non-oral consonants. Although outcomes are highly dependent on the surgeon’s experience, it would appear that early closure of the hard palate improves some language outcomes of children with UCLP.

Some authors have analyzed Scandcleft outcomes at 5 years and 10 years.

Willadsen et al. concluded that at 5 years of age, early closure (at 12 months) of the hard palate was the most effective in ensuring consonantal competence, whereas velopharyngeal function showed no significant improvement [77]. At 10 years, 58% of the children showed a PCC score equal to peers but with frequent sound distortions /s/, while 30% received secondary pharyngeal surgery, which, however, had little impact on language improvement.

Persson et al. concluded that the PCC score at 5 years was the main predictor of achieving language proficiency equal to peers at 10 years [93]. Speech therapy visits improved PCC scores, but were not decisive in achieving language competence equal to peers at 10 years. A high number of speech therapy visits did not lead to significant improvements, suggesting that the quality of the visits was more important than the number of visits.

The studies consistently concluded that early surgical treatment (within the first year) of the hard palate results in better language outcomes. Patients treated within the first year demonstrated substantial improvements in consonantal competence and speech intelligibility, achieving language competence comparable to their peers without CP. Though velopharyngeal function was unaffected by the timing of surgery, the surgeon’s expertise played a significant role in improving phonatory recovery.

## 5. Conclusions

This systematic review has highlighted the importance of choosing an appropriate surgical protocol for improving the speech of children with CP.

The VPI of children with CP causes alterations in nasal resonance and intelligibility. The management of these language abnormalities requires the early treatment of CP, preferably carried out within the first 12 months of life, the period of language development.

Among the surgical techniques analyzed, Sommerlad’s palatoplasty and Von Langenbeck’s technique proved effective in correcting CP; however, language outcomes were strongly influenced by the size of the defect and postoperative complications such as FONs and VPIs. Furlow’s double-opposite Z-plasty technique and modified V-Y palatoplasty were more complex to perform, but showed superior results in terms of soft palate mobility and reduction in hypernasality, resulting in significant improvements in intelligibility. Such techniques require a high degree of surgical expertise, suggesting the importance of a highly specialized approach.

Furthermore, studies have revealed that the repair of the hard palate within the first year of life is critical to ensuring improved PCC scores and intelligibility. Two-stage repair did not significantly affect long-term language outcomes compared to single-stage repair.

In conclusion, the studies confirmed that choosing an appropriate surgical technique and performing the repair surgery within the first 12 months of life are essential to the improvement in language in CP patients and the reduction in the need for secondary interventions to correct phonatory function.

While the current evidence does not allow for definitive comparisons across all surgical techniques, the Furlow palatoplasty and the V-Y pushback technique tend to demonstrate favorable outcomes in selected parameters such as soft palate mobility and reduction in hypernasality. These trends should, however, be interpreted with caution, as most studies do not include standardized assessments of muscle reconstruction or long-term speech outcomes. Future high-quality, randomized controlled trials with detailed surgical descriptions are needed to establish clearer guidelines.

### 5.1. Limitations of the Study

The primary limitation of this study is the heterogeneity of the included studies regarding the sample size, follow-up periods, and surgical techniques. The lack of standardized assessment protocols for speech outcomes poses challenges in comparing results across studies. Furthermore, the small sample sizes in certain studies limit the generalizability of the findings.

### 5.2. Future Objectives

Future research should aim to establish standardized protocols for evaluating speech outcomes following cleft palate surgery. Prospective multicenter studies with larger sample sizes are needed to determine the long-term efficacy of different surgical techniques. Additionally, exploring the integration of advanced imaging technologies and genetic profiling could provide more personalized treatment strategies.

## Figures and Tables

**Table 1:** A descriptive item selection summary.

Author	Type of Study	Outcome	Surgical Technique	Material and Methods	Results
Ha et al. (2015) [91]	Retrospective clinical study	To evaluate the clinical outcomes of primary cleft palate surgery, focusing on oronasal fistula rate, velopharyngeal insufficiency (VPI), and speech outcomes.	Furlow Z-plasty, two-flap palatoplasty, intravelar veloplasty (IVVP)	292 patients with nonsyndromic cleft palate (±cleft lip) underwent primary palatoplasty at Seoul Asan Medical Center (2007–2012). Techniques included Furlow Z-plasty, two-flap palatoplasty, and intravelar veloplasty. Follow-up speech assessments were conducted at 12, 15, 24, and 36 months.	Oronasal fistula rate: 7.9%. VPI requiring secondary surgery: 19.2%. Speech therapy required in 50.3% of cases. Hypernasality in 28.8%, and articulatory deficits in 51.4%. Cleft type influenced speech outcomes and VPI rates.
Kappen et al. (2017) [92]	Retrospective follow-up study	To evaluate long-term speech outcomes in adults with unilateral complete cleft lip and palate (UCLP) treated with two-stage palatoplasty.	Two-stage palatoplasty (soft: IVVP, hard: Von Langenbeck) + pharyngoplasty if needed	48 adults with UCLP who underwent two-stage palatal closure were assessed for speech outcomes. Medical history was reviewed, and speech evaluations were performed by a speech therapist. Nasometry and intelligibility scales were used.	84.4% had intelligible speech (scores 1–2 on a 5-point scale). A total of 36% showed mild to moderate hypernasality. A total of 40% underwent pharyngoplasty. Higher incidence of articulation errors correlated with lower intelligibility scores.
Persson et al. (2024) [93]	Longitudinal study	Analyze speech development in children with unilateral cleft lip and palate (UCLP) at ages 5 and 10, evaluating the role of speech therapy and secondary velopharyngeal surgery.	Based on multicenter protocols	Data analysis from the Scandcleft project, including 320 children from five countries. Speech proficiency was assessed using velopharyngeal competence (VPC-Sum) and the percentage of consonants correct (PCC). The number of speech therapy sessions and secondary surgeries were also considered.	At age 5, only 23% of children had speech proficiency at “peer level,” increasing to 56% by age 10. The best predictor of speech competence at age 10 was proficiency at age 5. A high number of speech therapy sessions did not significantly improve outcomes.
Hammarström et al. (2020) [94]	Prospective RCT	Compare speech outcomes in 1-stage vs. 2-stage palatal closure in 5-year-olds with UCLP.	1-stage vs. 2-stage palatal closure	112 children (5 years old) from Sweden and Finland. Arm A: soft palate closure at 4 months, hard palate at 12 months. Arm C: both closures at 12 months. Evaluations: VPC, PCC, consonant errors, speech therapy visits.	No significant differences in VPC/VPI between groups. PCC scores were generally low, with Finnish center showing better results. Swedish centers had more consonant errors and speech therapy visits.
Persson et al. (2020) [95]	Prospective RCT	Compare speech outcomes in 2-stage palatal closure sequencing in 5-year-olds with UCLP.	Based on multicenter protocols	136 children (5 years old) from Norway and UK. Arm A: lip and soft palate closure at 3–4 months, hard palate at 12 months. Arm D: lip and hard palate closure at 3–4 months, soft palate at 12 months. Evaluations: VPC, PCC, CSCs, and speech therapy visits.	No significant differences in VPC or PCC between groups. Some centers showed higher CSCs in Arm A. Wide variability in speech outcomes across centers.
Willadsen et al. (2017) [77]	Randomized controlled trial (RCT)	Evaluate speech outcomes in children with UCLP based on timing of hard palate closure.	Hard palate closure at 12 vs. 36 months	143 children (5 years old), hard palate closure at 12 months (arm A) or 36 months (arm B), assessed using VPC, PCC, and speech therapy visits.	PCC scores ranged from 86 to 92%. VPC was achieved in 58–83% of participants. No single protocol proved superior. Girls had better speech outcomes. Factors like hearing level, speech therapy, and secondary surgeries influenced results.
Willadsen et al. (2019) [76]	Longitudinal randomized controlled trial (RCT)	To evaluate the development of obstruent correctness and speech error types in Danish children with unilateral cleft lip and palate (UCLP) from ages 3 to 5.	Hard palate closure at 12 vs. 36 months	108 children with UCLP from the Scandcleft Project received early (12 months) or late (36 months) hard palate closure. Speech recordings at ages 3 and 5 were transcribed phonetically by blinded raters. Analyzed error types included cleft speech characteristics (CSCs) and developmental speech characteristics (DSCs).	PCC-obs scores improved from ages 3 to 5, but children with UCLP did not reach typical Danish children’s speech levels. Late closure group had significantly lower scores at age 5 than early closure group at age 3. CSCs at age 3 strongly predicted PCC-obs at age 5.
Bruneel et al. (2018) [96]	Retrospective cohort study	To evaluate speech outcomes following Sommerlad primary palatoplasty and compare them with an age- and gender-matched control group.	Sommerlad	16 patients with cleft palate (mean age: 5.4 years) treated at Ghent University Hospital. Speech intelligibility, resonance, nasal airflow, and articulation were assessed perceptually. Nasalance values and NSI 2.0 were measured instrumentally.	The CPLE toothpaste/mouthwash provides an effective and natural alternative to SLS-free toothpaste +/− mouthwash containing EO when used as a complement to mechanical oral hygiene to reduce interdental gingival inflammation.
Jørgensen et al. (2020) [97]	Longitudinal study (RCT sub-study)	Evaluate obstruent correctness (PCC-obs) development and error patterns in Danish children with unilateral cleft lip and palate (UCLP) from ages 3 to 5, identifying predictors of PCC-obs at age 5.	Early vs. late hard palate closure (12 vs. 36 mo)	Analyzed data from the Scandcleft Project, including 108 Danish children with UCLP who underwent either early hard palate closure (EHPC at 12 months) or late hard palate closure (LHPC at 36 months). Phonetic transcription of speech samples from naming tests at ages 3 and 5 was performed by blinded raters.	PCC-obs significantly improved from ages 3 to 5, with greater gains in the LHPC group. However, at age 5, the LHPC group still did not reach the PCC-obs level of the EHPC group at age 3. Higher CSC and DSC frequencies at age 3 predicted lower PCC-obs at age 5. VPD and gender had minimal predictive impact.
Mark et al. (2023) [98]	Longitudinal follow-up study	To analyze speech outcomes in individuals with UCLP after Gothenburg two-stage palate closure (soft palate at 6 months, hard palate at 3 years).	Two-stage (soft at 6 months, hard at 3 years)	28 patients underwent a two-stage closure. Speech samples recorded at 5, 10, 16, and 19 years were evaluated by three independent speech–language pathologists. Variables included hypernasality, articulation errors, and velopharyngeal function.	25–30% of participants showed articulation disorders at 5 years, but most resolved later. Velopharyngeal incompetence in 20% at 5 years but none at 19 years. Fewer articulation errors compared to patients with later hard palate closure at 8 years.
Chapman et al. (2024) [99]	Prospective, longitudinal, observational, comparative effectiveness study	To compare speech outcomes and fistula rates between two palate repair techniques (IVVP vs. Furlow Z-plasty), evaluate early intervention speech–language (EI-SL) services, and analyze their impact on speech outcomes.	IVVP vs. Furlow Z-plasty	1247 children with cleft palate (CP ± L) were enrolled from 20 sites in the US. Exclusion criteria included submucous cleft palate and bilateral sensorineural hearing loss. Primary outcome: perceptual ratings of hypernasality at age 3. Secondary outcomes: fistula rate, speech production, and quality of life. Statistical analysis used generalized estimating equations with propensity score weighting.	Recruitment completed in 2023 with 80% retention. In total, 562 children completed the final 3-year speech assessment. Final study activities to conclude in 2025. The study aims to resolve uncertainties about the effectiveness of IVVP vs. Furlow Z-plasty and improve cleft care research.
Hofman et al. (2024) [100]	Retrospective cohort study	To assess long-term speech outcomes and incidence of velopharyngeal insufficiency (VPI) after Sommerlad palatoplasty.	Sommerlad palatoplasty	239 patients with cleft lip and/or palate (CL/P) treated at Wilhelmina Children’s Hospital (2008–2017) were reviewed. Inclusion criteria: Sommerlad palatoplasty, speech assessment at age 5 or older. Outcomes analyzed using chi-squared tests and odds ratios.	VPI rate: 52.7%. Speech correction surgery required in 49.8%. Higher Veau classification and cleft width >10 mm were significantly associated with worse speech outcomes. Fistula presence increased the likelihood of additional surgery.
Oyama et al. (2016) [101]	Retrospective cohort study	To compare speech, nasometric, and cephalometric outcomes after modified V-Y palatoplasty with or without a mucosal graft.	Modified V-Y palatoplasty with/without mucosal graft	191 patients underwent primary palatoplasty (82 with mucosal graft, 109 without). Speech assessments included hypernasality rating, nasal emission, and nasometry. Cephalometric analysis evaluated velopharyngeal morphology and velar movement.	Normal resonance was significantly higher in the mucosal graft group (90.3%) vs. non-graft group (68.8%). Mean nasalance scores were lower in the graft group, approaching control levels. Cephalometry showed greater velar length and elevation in the graft group.
Brudnicki et al. (2023) [102]	Retrospective case–control study	To assess speech outcomes and the burden of secondary surgical interventions in UCLP patients undergoing one-stage repair and alveolar bone grafting at different ages.	Double-layer vomeroplasty; early vs. late ABG	56 patients with unilateral cleft lip and palate (UCLP) were divided into early (<6 years) and late (>6 years) alveolar bone grafting (ABG) groups. Speech assessments were performed at age 10 using 27 standardized sentences. Secondary surgical interventions, such as pharyngoplasty and fistula repair, were recorded.	7 patients had disordered speech intelligibility. A total of 12 had hypernasality, 13 had nasal emission, and 5 had nasal turbulence. Speech outcomes were significantly worse than the control group. A Dutch speech assessment protocol will be developed to standardize evaluations.

## Data Availability

Not applicable.

## Abbreviations

The following abbreviations are used in this manuscript:

OFC	Orofacial cleft
CL	Cleft lip
CP	Cleft palate
CL/P	Cleft lip and palate
VPI	Velopharyngeal insufficiency
IVVP	Intravelar veloplasty
PCC	Percent consonant correct
FON	Oronasal fistulas
NSI	Nasal Severity Index
UCLP	Unilateral cleft lip and palate
RCT	Randomized controlled trial
